# Impact of low-flow desflurane versus sevoflurane anesthesia on thiol–disulfide homeostasis in adults undergoing elective thyroidectomy: a randomized controlled trial

**DOI:** 10.1590/1806-9282.20250893

**Published:** 2026-03-30

**Authors:** Elzem Sen, Alper Aytekin

**Affiliations:** 1 Gaziantep University, Faculty of Medicine, Department of Anesthesiology and Reanimation – Gaziantep, Turkey.; 2 Gaziantep University, Faculty of Medicine, Department of General Surgery – Gaziantep, Turkey.

**Keywords:** Anesthesia, inhalation, Desflurane, Sevoflurane, Oxidative stress, Disulfides

## Abstract

**OBJECTIVE:**

Thiol–disulfide homeostasis is a marker of oxidative stress. The aim of the study was to compare the effects of low-flow desflurane and sevoflurane anesthesia on perioperative changes in thiol–disulfide balance in patients undergoing thyroidectomy.

**METHODS:**

In this randomized controlled trial, 50 ASA I–II patients were assigned to receive low-flow desflurane (n=25) or sevoflurane (n=25). Blood samples were collected before and after surgery. The primary outcomes were between-group comparisons of perioperative changes in total and native thiol levels. Secondary outcomes included changes in disulfide concentrations and thiol–disulfide ratios.

**RESULTS:**

For the primary outcomes, the magnitude of reduction in total thiol (86.4±159.7 vs. 151.5±104.9 μmol/L; p=0.095) and native thiol (79.3±130.1 vs. 115.1±86.3 μmol/L; p=0.257) did not differ significantly between the desflurane and sevoflurane groups. For the secondary outcomes, disulfide levels significantly decreased in the sevoflurane group (67.5±19.9 vs. 48.4±18.1 μmol/L; p=0.001), whereas no significant change was observed in the desflurane group (60.2±23.2 vs. 52.5±34.5 μmol/L; p=0.300). Between-group comparisons of disulfide/total thiol, native thiol/total thiol, and disulfide/native thiol ratios revealed no significant differences.

**CONCLUSION:**

Low-flow desflurane and sevoflurane anesthesia demonstrated similar effects on perioperative thiol–disulfide homeostasis in thyroidectomy patients.

## INTRODUCTION

Low-flow anesthesia, a semi-closed inhalational technique, is increasingly used due to its economic and environmental advantages. Modern anesthesia workstations have improved its safety, supporting widespread adoption^1,2^.

Oxidative stress is a key concern during surgery, and thiol– disulfide homeostasis (TDH) serves as a sensitive biomarker of redox balance. Alterations in thiol and disulfide levels reflect the oxidative burden and antioxidant capacity of the body. While the cytotoxic and oxidative effects of volatile anesthetics have been documented, their specific impact on TDH under low-flow conditions remains unclear^3,4^.

To address this gap, we designed a randomized controlled trial to compare the effects of low-flow desflurane and sevoflurane anesthesia on TDH in patients undergoing thyroidectomy. We hypothesized that these agents would exert differential effects, with sevoflurane potentially demonstrating more favorable antioxidant properties.

## METHODS

After obtaining Local Ethical Committee approval (Date: 23.09.2020, No: 2020/283), this prospective, parallel-group, randomized controlled trial was carried out at our hospital from September 2020 until January 2021. The principles of the Declaration of Helsinki were obeyed at all stages. We report this trial in accordance with CONSORT 2010. In line with ICMJE transparency recommendations, trial registration details are provided; registration was retrospective (ClinicalTrials.gov NCT06810453; first posted 14 January 2025), and no post-hoc changes were made to prespecified outcomes or the statistical analysis plan.

Fifty ASA (American Society of Anesthesiologists) physical status I–II adult patients scheduled for elective total thyroidectomy under general anesthesia were enrolled and provided written informed consent. Patients with diabetes mellitus, alcohol or drug addiction, smoking habits, expected difficult intubation, morbid obesity, chronic obstructive pulmonary disease, cardiovascular disease, and those in pregnancy or lactation period were excluded from the study. Demographic data [age, gender, body mass index (BMI)] and ASA physical status of the patients were recorded.

Patients were randomly allocated (1:1 ratio) using a computer-generated sequence into two groups: Group D (low-flow desflurane) and Group S (low-flow sevoflurane). Allocation concealment was ensured using sealed, opaque, sequentially numbered envelopes opened by the study coordinator only after patient enrollment. Both patients and outcome assessors were blinded to group allocation. Due to the nature of the intervention, care providers were not blinded but followed a standardized protocol to minimize bias.

Standardized anesthesia protocols were followed in both groups, ensuring uniformity in perioperative management. Premedication was not administered, and upon admission to the operating room, 5 cc blood samples were collected to evaluate TDH after intravenous access was established. Subsequently, preoxygenation was performed with 100% oxygen at a flow rate of 10 L/min through a face mask for 3 min. Standard hemodynamic monitoring included continuous pulse oximetry, electrocardiogram, and blood pressure measurements every three minutes. General anesthesia was induced with intravenous fentanyl (2 mcg/kg), propofol (2 mg/kg) and rocuronium (0.6 mg/kg). Then, endotracheal intubation was carried out, and volatile anesthetics were initiated once the patients were connected to the ventilator. An anesthesia workstation with an electronic vaporizer (Aisys Carestation, GE Healthcare, Madison, WI, USA) was used. Anesthesia maintenance included desflurane end-tidal at 4.5–5.0% or sevoflurane end-tidal concentration at 1.2–1.4%, both at a flow rate of 1 L/min and a dynamic adjustment of remifentanil infusion (0.2–0.8 mcg/kg/min). Ventilator parameters such as tidal volume (6–8 mL/kg) and respiratory rate were adjusted to maintain end-tidal CO_2_ within the range of 30–40 mmHg according to standard department guidelines. A 5 cc venous blood sample was obtained at the end of surgery. Volatile anesthetic agents were discontinued nearly 10 min before extubation, and decurarization was provided by 2 mg/kg sugammadex. When sufficient muscle strength and airway stability were obtained, they were taken to the postoperative recovery unit.

### Collection of materials

Blood samples were collected from volunteers in tubes containing gel serum tubes (Becton Dickinson, UK). Blood samples, which were placed in gel serum tubes, were allowed to coagulate for about 20 min at room temperature. After coagulation, blood samples were centrifuged for 10 min at 4,000 rpm. Serum samples were stored at -80°C until the study day.

### Serum thiol–disulfide homeostasis parameters measurement/calculation

Serum TDH parameters were evaluated using an automated method^3^. Homeostasis parameters include disulfide, native thiol (NT) and total thiol (TT) levels, and disulfide/TT, NT/TT, and disulfide/NT ratios. TT and NT levels were measured in μmol/L on a Beckman Coulter AU480 analyzer using a commercial kit (Rel Assay Diagnostics, Turkey). The amount of disulfide was calculated using the formula [(TT-NT)/2]. Then, ratios were calculated as percentages^5,6^.

The primary outcome measure of this study was the effect of low-flow desflurane and sevoflurane anesthesia on TDH, assessed by measuring preoperative and postoperative total thiol and native thiol values. The secondary outcome measures included preoperative and postoperative disulfide levels, native thiol/total thiol ratios, disulfide/total thiol ratios, and disulfide/native thiol ratios.

### Power analysis

The effect size assumption for our power analysis was derived from the study by Ozcan et al., who observed significant changes in thiol–disulfide parameters with volatile anesthetics. Based on those reported mean differences in total thiol levels, we calculated that a sample size of 25 patients per group would provide 80% power to detect a clinically meaningful difference at α=0.05. To further support the interpretation of results, effect sizes were calculated using Cohen’s d for both within-group and between-group comparisons. Additionally, 95%CIs for mean differences were provided for the primary outcome variables.

### Statistical analysis

Normality was assessed using the Shapiro-Wilk test. All continuous variables were normally distributed and are presented as mean±standard deviation. Depending on data distribution, comparisons between two independent groups were made using Student’s t-test, while a paired t-test was used for repeated measures. Categorical variables were analyzed with the chi-square test. Sensitivity analysis of covariance (ANCOVA) was performed, adjusting for baseline values and height with treatment as the fixed factor. Assumptions were met; results are reported as adjusted mean differences (95%CI, two-sided p). Analyses were conducted using Statistical Package for the Social Sciences (SPSS) v22.0, with p<0.05 considered statistically significant.

## RESULTS

**Figure 1:**
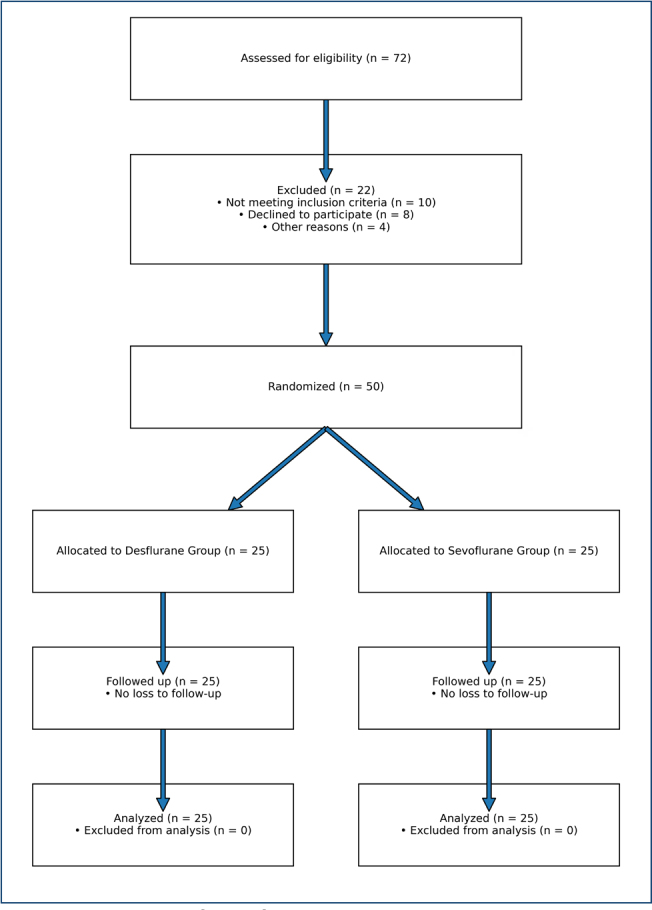
CONSORT flow of the participants.

A total of 50 patients were included in the analysis, with 25 patients in Group D (low-flow desflurane) and 25 patients in Group S (low-flow sevoflurane). The flow of patient enrollment, randomization, and allocation is presented in the CONSORT diagram (Figure 1). No intraoperative or postoperative complications were observed in either group.

Baseline demographic characteristics are presented in Table 1. There was no significant difference between the groups regarding age (40.9±12.8 vs. 45.2±12.2 years; p=0.231) or gender distribution (p=0.637). Mean body weight (79.9±17.5 vs. 79.9±13.6 kg; p=0.986) and BMI (30.7±5.9 vs. 28.3±4.3 kg/m^2^; p=0.108) were comparable. A significant difference was observed in height (161.4±6.6 vs. 168.0±7.1 cm; p=0.001), although correlation analysis demonstrated no association between height and oxidative stress parameters. The duration of surgery and ASA scores were similar between the groups (p=0.173 and p=0.549, respectively).

**Table 1:** Demographic characteristics of groups.

Variables	Group D (n=25) Mean±SD	Group S (n=25) Mean±SD	p-value
Age (years)	40.88±12.75	45.16±12.19	0.231
Gender n (%)			
Female	22 (88%)	23 (92%)	0.637
Male	3 (12%)	2 (8%)	
Weight (kg)	79.96±17.49	79.88±13.57	0.986
Height (cm)	161.36±6.58	168.04±7.12	0.001^*^
BMI (kg/m^2^)	30.65±5.88	28.25±4.33	0.108
Duration of operation (minutes)	176.40±48.83	161.16±25.47	0.173
ASA score n (%)			
1	0 (0%)	1 (4%)	
2	21 (84%)	19 (76%)	0.549
3	4 (16%)	5 (20%)	

*p<0.05, BMI: body mass ındex, SD: standard deviation, ASA: American Society of Anaesthesiologists; Although height differed between groups, it showed no correlation with oxidative stress parameters and is unlikely to affect outcomes.

As presented in Table 2, preoperative total thiol levels were significantly lower in Group D compared with Group S (654.9±117.8 vs. 714.9±78.4 μmol/L; p=0.039; Cohen’s d=-0.60, 95%CI -1.17 to -0.03). Postoperatively, there was no statistically significant between-group difference in total thiol (568.4±142.5 vs. 563.4±106.2 μmol/L; p=0.888; d=0.04, 95%CI -0.53 to 0.61), and the wide CI indicates limited precision. Within-group analysis revealed significant decreases from preoperative to postoperative values in both Group D (p=0.012) and Group S (p=0.006). The between-group difference in the magnitude of reduction did not reach statistical significance (86.4±159.8 vs. 151.5±104.9 μmol/L; p=0.095; d=-0.48, 95%CI -1.05 to 0.09).

**Table 2:** Preoperative and postoperative total thiol and native thiol values and their correlation with height.

Variables	Group D (n=25) Mean±SD	Group S (n=25) Mean±SD	p-value	Cohen’s d (Group D–Group S)	95%CI	r with height	p (corr)
Preop total thiol (μmol/L)	654.85±117.84	714.94±78.37	0.039^*^	-0.60	[-1.17, -0.03]	-0.004	0.978
Postop total thiol (μmol/L)	568.44±142.49	563.4±106.17	0.888	0.04	[-0.53, 0.61]	-0.112	0.442
p	0.012^*^	0.006^*^					
Difference	86.41±159.75	151.54±104.89	0.095	-0.48	[-1.05, 0.09]		
Preop native thiol (μmol/L)	536.6±83.14	578.15±46.06	0.034^*^	-0.62	[-1.19, -0.05]	0.047	0.746
Postop native thiol (μmol/L)	457.29±112.91	463.02±88.29	0.843	-0.06	[-0.63, 0.51]	-0.108	0.457
p	0.001^*^	0.001^*^					
Difference	79.31±130.12	115.14±86.25	0.257	-0.32	[-0.89, 0.24]		

*p<0.05; Student’s t-test was used for between-group comparisons and paired t-test for within-group comparisons. All variables were normally distributed; therefore, data are presented as mean±SD. Effect sizes were calculated using Cohen’s d (Group D–Group S), with 95%CI. Negative d values indicate lower means in Group D relative to Group S. Pearson correlation analysis showed no significant association between height and oxidative stress parameters (p>0.05). SD: standard deviation; CI: confidence interval.

Similarly, preoperative native thiol levels were significantly lower in Group D than Group S (536.6±83.1 vs. 578.2±46.1 μmol/L; p=0.034; d=-0.62, 95%CI -1.19 to -0.05). Postoperative native thiol levels did not differ significantly (457.3±112.9 vs. 463.0±88.3 μmol/L; p=0.843; d=-0.06, 95%CI -0.63 to 0.51). Within-group reductions were significant in both Group D (p=0.001) and Group S (p=0.001), but the intergroup difference in change did not reach significance (79.3±130.1 vs. 115.1±86.3 μmol/L; p=0.257; d=-0.32, 95%CI -0.89 to 0.24). **Correlation analysis did not show a statistically significant association between height and oxidative stress parameters** (p>0.05). In ANCOVA adjusted for baseline value and height, there were no significant between-group differences for total thiol (-26.6 μmol/L; 95%CI -109.1 to 55.8; p=0.519), native thiol (-11.3; -79.2 to 56.7; p=0.740), or disulfide (-7.46; -25.27 to 10.35; p=0.404); height was unrelated to outcomes, and there was no interaction between group and height.

Disulfide levels and thiol–disulfide ratios are summarized in Table 3. Preoperative and postoperative disulfide concentrations were not significantly different between the groups (60.2±23.2 vs. 67.5±19.9 μmol/L, p=0.225; and 52.5±34.5 vs. 48.4±18.1 μmol/L, p=0.749). Within-group analysis demonstrated a significant postoperative reduction in disulfide levels in Group S (p=0.001) but not in Group D (p=0.300). The reduction was not statistically significant (p=0.077; d=-0.81, 95%CI -1.38 to -0.22); the estimate suggests a larger decrease in Group S, but the CI indicates uncertainty.

**Table 3:** Preoperative and postoperative disulfide levels and thiol–disulfide ratios.

Variables	Group D (n=25) Mean±SD	Group S (n=25) Mean±SD	Between-group p	Cohen’s d (D–S)	95%CI for d	Notes
Disulfide (μmol/L)—preoperative	60.20±23.19	67.52±19.85	0.225	-0.34	[-0.90, 0.22]	
Disulfide (μmol/L)—postoperative	52.50±34.48	48.37±18.07	0.749	0.15	[-0.41, 0.70]	
Disulfide (μmol/L)—difference (Post–Pre)	4.87±16.70	18.17±16.31	0.077	-0.81	[-1.38, 0.76]	Within-group p (D/S): 0.300/0.001^*^
Native thiol/total thiol (%)—preoperative	82.05±4.51	81.33±1.97	0.567	0.21	[-0.35, 0.76]	
Native thiol/total thiol (%)—postoperative	82.71±5.33	83.60±2.86	0.720	-0.21	[-0.76, 0.35]	
Native thiol/total thiol (%)—difference (Post–Pre)	-0.32±4.69	-2.18±1.92	0.399	0.52	[-0.05, 1.08]	Within-group p (D/S): 0.989/0.166
Disulfide/total thiol (%)—preoperative	9.15±2.25	9.34±1.47	0.567	-0.10	[-0.65, 0.46]	
Disulfide/total thiol (%)—postoperative	8.97±2.64	8.87±1.99	0.720	0.04	[-0.51, 0.60]	
Disulfide/total thiol (%)—difference (Post–Pre)	0.79±2.37	1.21±1.48	0.399	-0.21	[-0.77, 0.34]	Within-group p (D/S): 0.989/0.166
Disulfide/native thiol (%)—preoperative	11.25±2.44	11.51±1.67	0.567	-0.12	[-0.68, 0.43]	
Disulfide/native thiol (%)—postoperative	10.42±2.88	9.85±1.84	0.720	0.24	[-0.32, 0.79]	
Disulfide/native thiol (%)—difference (Post–Pre)	0.95±2.86	1.36±1.90	0.399	-0.17	[-0.72, 0.39]	Within-group p (D/S): 0.968/0.183

*p<0.05. Student’s t-test for between-group comparisons; paired t-test for within-group (Post vs. Pre). All variables were normally distributed. Effect sizes are Cohen’s d (Group D–Group S) with 95%CIs; negative values indicate lower means in Group D. SD: standard deviation; CI: confidence interval.

Thiol–disulfide ratios, including native thiol/total thiol, disulfide/total thiol, and disulfide/native thiol percentages, showed no significant between-group differences either pre-operatively or postoperatively. Within-group changes in these ratios were small and not statistically significant, and effect sizes were negligible.

## DISCUSSION

This study compared the effects of desflurane and sevoflurane under low-flow anesthesia on TDH, a marker of oxidative stress. Both agents, used during elective thyroidectomy, were associated with a decrease in native and total thiol levels and an increase in disulfide levels, consistent with disruption of TDH.

Surgical trauma can induce a stress response that increases free radical production. When antioxidant defenses are overwhelmed, oxidative stress occurs, potentially contributing to systemic inflammatory response syndrome^7^. General anesthesia, maintained with volatile agents like desflurane and sevoflurane, is essential for surgical procedures. These agents have antioxidant and anti-inflammatory effects but may also influence oxidative stress^8, 9, 10, 11, 12^.

Kaşıkara et al. demonstrated the effectiveness of low-flow sevoflurane in reducing oxidative damage during metabolism^1^. Additionally, Eminoglu et al. found no significant difference in thiol/disulfide homeostasis between low-flow and high-flow anesthesia techniques in their study^13^.

Plasma thiols, consisting mainly of protein-bound forms like albumin and smaller low-molecular-weight thiols, are essential for key biological functions. They contribute to antioxidant defense, detoxification, apoptosis, enzyme activity regulation, and cellular signaling by acting through their sulfur-containing chemical structure^14,15^.

Reactive oxygen species (ROS) primarily target sulfur-containing amino acids like cysteine and methionine, oxidizing protein thiols into disulfides. This reversible thiol–disulfide exchange highlights protein thiols as key in vivo targets of ROS and reflects the activity of the body’s antioxidant defense mechanisms^16^. Dynamic TDH reflects oxidative stress and can help monitor disease progression and treatment response^17^. Total thiols include both reduced and oxidized forms, while native thiols represent only the reduced form. Elevated ROS levels can oxidize cysteine residues, shifting TDH toward disulfide formation^18^. Akin et al. indicated that antioxidants containing thiols are the first components to act against increased ROS during surgery or anesthesia^18^.

Our results differ from those of Ozcan et al., who found sevoflurane to exert superior antioxidant effects compared with desflurane during laparoscopic cholecystectomy. This discrepancy may be explained by differences in the type and extent of surgical trauma (thyroidectomy vs. laparoscopic cholecystectomy), patient characteristics, and baseline oxidative stress levels. Furthermore, while we observed a significant within-group decrease in disulfide levels with sevoflurane, the lack of significant between-group differences was not statistically significant, and CIs were wide, so modest agent-related differences cannot be excluded under low-flow conditions^4^.

Kutluhan et al. found that thiol–disulfide homeostasis reflects oxidative stress in patients undergoing vertebral surgery. Propofol reduced oxidative stress more effectively than desflurane. Both groups showed decreased total and native thiol levels postoperatively^17^. Kazancioglu et al. investigated the effects of hypotensive anesthesia with desflurane during elective septoplasty and found that it did not negatively affect thiol-disulfide balance, suggesting it is safe in terms of oxidative stress response^19^.

Previous research showed that dexmedetomidine reduced oxidative stress markers and improved TDH in cardiac surgery patients. These findings highlight the potential antioxidant benefits of combining dexmedetomidine with volatile anesthetics, warranting further investigation in various surgical contexts^20^.

We did not detect statistically significant between-group differences in total and native thiol levels; the sevoflurane group showed a within-group reduction in disulfide, but the between-group comparison was not significant, and estimates were imprecise.

Thiol–disulfide imbalance is associated with increased oxidative stress, which may contribute to postoperative complications such as delayed recovery, impaired wound healing, or systemic inflammatory response^21^. Although our study did not evaluate clinical endpoints, the absence of statistically significant between-group differences in thiol–disulfide homeostasis should be interpreted cautiously, because small effects cannot be excluded. Future research integrating biochemical markers with clinical parameters such as recovery profiles, pain, and complication rates is needed to clarify the relevance of these biochemical changes for patient care.

The study was not powered to detect small between-group differences, and several confidence intervals were wide; therefore, non-significant findings should be interpreted with caution. We emphasize effect sizes with 95%CIs rather than post-hoc power and note that with 25 participants per group, our design provides adequate power primarily for moderate-to-large effects, whereas observed effects were smaller.

### Limitations

The relatively small sample size (n=50) may have limited the ability to detect subtle but clinically relevant differences, as reflected by wide confidence intervals. Although outcome-assessor blinding was ensured, care providers could not be blinded, which may have introduced performance bias. Baseline height imbalance was present; however, sensitivity analyses using ANCOVA (adjusting for baseline values and height) yielded results consistent with the primary analyses, indicating that this imbalance did not materially affect outcomes. The retrospective trial registration represents another limitation, although predefined outcomes and randomization with assessor blinding helped reduce bias. This study was designed as a biochemical comparison and did not assess clinical outcomes (e.g., recovery time, complications, hospital stay). Future studies should link thiol–disulfide markers to patient-centered endpoints. Institutional variations in perioperative care may also influence oxidative stress markers. Despite these limitations, the randomized design and the focus on oxidative stress in low-flow anesthesia strengthen the study.

## CONCLUSION

In conclusion, low-flow desflurane and sevoflurane anesthesia demonstrated broadly similar effects on thiol–disulfide homeostasis in patients undergoing thyroidectomy. Although a greater reduction in disulfide levels was observed in the sevoflurane group, the between-group difference was not statistically significant. Larger, multicenter studies are warranted to clarify whether this biochemical trend has clinical relevance.

## Data Availability

The datasets generated and/or analyzed during the current study are available from the corresponding author upon reasonable request.
